# Mothers’ knowledge, attitude and practice towards the prevention and home-based management of diarrheal disease among under-five children in Diredawa, Eastern Ethiopia, 2016: a cross-sectional study

**DOI:** 10.1186/s12887-018-1321-6

**Published:** 2018-11-19

**Authors:** Hailemariam Mekonnen Workie, Abdilahi Sharifnur Sharifabdilahi, Esubalew Muchie Addis

**Affiliations:** School of Nursing and Midwifery, College of Health and Medical Science, Haramaya University, P.O. Box 235, Harar, Ethiopia

**Keywords:** Knowledge, Attitude, Practice, Mothers, Prevention, Home-based management, Diarrhea, Under-five children

## Abstract

**Background:**

Diarrhea remains the 2nd leading cause of death among children under 5 globally. It kills more young children than AIDS. It would have been prevented by simple home management using oral rehydration therapy. Mothers play a central role in its management and prevention. So, the main objective of this study was to assess mothers’ knowledge, attitude & practice in prevention & home-based management of diarrheal disease among under-five children in Dire Dawa, Eastern Ethiopia.

**Methods:**

Institutional based cross-sectional study was conducted from March 15–April 14, 2016, in Diredawa among 295 Mothers who had under-five child with diarrhea in the last 2 weeks using simple random sampling method. Mothers were interviewed face to face by using pretested, standard and structured questionnaire. The data quality was assured by translation, retranslation and pretesting the questionnaire. Data were checked for completeness, consistency and then entered into Epi Info v3.1 and analyzed using SPSS v20. The descriptive statistical analysis was used to compute frequency, percentages, and mean of the findings of this study. The results were presented using tables, charts, and graphs.

**Results:**

In this study, 295 participants were included with 100% response rate. From total 295 mothers, around two-thirds (65.2%) of them had good knowledge, but more than half of mothers (54.9%) had a negative attitude towards home-based management and prevention of diarrhea among under-five children. Regarding the attitude of the mothers, 58% had poor practice towards home-based management and prevention of diarrhea among under-five children.

**Conclusion:**

The finding of this study showed that the attitude and practice of mothers were unsatisfactory about the prevention and home-based management of under-five diarrheal diseases. Therefore, Health education, dissemination of information, and community conversation should plan and implement to create a positive attitude and practice towards the better prevention and management of under 5 diarrheal diseases.

**Electronic supplementary material:**

The online version of this article (10.1186/s12887-018-1321-6) contains supplementary material, which is available to authorized users.

## Introduction

According to WHO, Passage of 3 or more than 3 loose of stool or watery stools per day or considers as abnormal by the mothers or stools more frequent than normal for a child is considered as diarrhea [1, 2]. Diarrheal disease remains the second leading cause of death among under 5 children globally [3–6]. Nearly one in five deaths of a child – about 1.5 million each year – is due to the disease of diarrhea [4, 7]. It kills more young children than malaria HIV/AIDS, and measles together [1, 4].

Diarrheal disease is one of the commonest illnesses that has the greatest negative impact on the growth and development of infants and young children [8]. Worldwide, children whose age is less than 5 years’ experience, on average, 3.2 episodes of diarrhea every year and consequently 1.87 million children will die from dehydration associated with diarrheal disease, particularly in the countries of Asia, Africa and Latin America [3].

According to Ethiopian demographic health survey (EDHS) of 2000, 2005, 2011 and 2016 the 2 weeks prevalence of diarrheal disease among under-five children was 24, 18, 13, 12% respectively [9–12]. Even though there was a double reduction of the prevalence of under 5 diarrheal diseases in the last 16 years in Ethiopia, but, still it is one of the most important public issue and major health problems of the country [9, 12].

Rotavirus is among the commonest diarrheal pathogen in children worldwide that causes about one-third of diarrhea-associated hospitalizations and 800,000 deaths per year [13–15]. Children in the poorest countries like Ethiopia account for 82% of rotavirus deaths of under-five children [16]. Rotavirus can cause intestinal losses of fluid, electrolyte and nutritional deficiency which relatively progresses rapidly to cause dehydration and death [17, 18].

Contaminated weaning food, inappropriate feeding practice, lack of clean water, poor hand washing, limited sanitary disposal of waste, poor housing conditions, and lack of access to adequate and affordable health care are aggravated factors of the under 5 diarrheal disease [6, 8, 19, 20].

Diarrheal diseases among under 5-year children can be tackled in at both primary and secondary prevention levels. The former about the improvement of sanitation and water quality but the latter is about early recognition of dehydration due to diarrhea and prompt oral rehydration using ORS (oral rehydration solution) or appropriate home available fluids. Oral rehydration solution has been proven to be effective in preventing diarrhea mortality in the community while varying degree of evidence favors the use of home available fluid [21].

Optimal infant & young child feeding practices could prevent more than 10% of deaths from diarrhea. On the other hand, better hygiene practices, particularly hand washing with soap & the safe disposal of excreta can reduce the incidence of diarrhea by 35% [1, 22].

Diarrhea is not lethal itself, the improper knowledge, poor practice and negative attitudes of mothers and their misdirected approach towards its management and prevention leads to high degree of severe dehydration and lastly death [23, 24]. Therefore, the main objective of this study was to assess the mothers’ knowledge, attitude, and practice in the prevention and home-based management of diarrhea towards their under-five children in Diredawa, East Ethiopia.

## Method

### Study area and period

The study was conducted from March 15 –April 14, 2016, in Diredawa city. Diredawa city is one of the two administrative cities in Ethiopia. It situated and located in the eastern part of Ethiopia with 515 km from Addis Ababa (capital city of Ethiopia) and 313 from Djibouti. According to the 2011 Ethiopian Demographic health survey (EDHS), the total population of the administration was 341,834 of which 174,461 were men and 170,461 women [11]. About 233,224 (68.23%) of the population were urban inhabitants, while 31.77% were rural inhabitants. In Dire-Dawa administration there was 2 governmental and 4 private hospitals. From these, the 3 hospitals were selected for this study.

### Study design and participants

A cross-sectional study design was conducted in selected Diredawa hospitals to assess mothers’ knowledge, attitude & practice towards the prevention & home-based management of diarrheal disease among under-five children. Mothers who had a child less than 5 years of age with diarrhea in the last 2 weeks were included in an interview using each hospital monthly patient flow report as a sampling frame. Those mothers with a physical impairment (unable to hear and speak) and mentally ill were excluded from the study.

### Sample size determination and technique

The sample size (n) required for this study was determined using a single population proportion formula (n = (Zα/2)^2^ p(1-p)/d^2^)); whereas n = the required sample size for this study, Zα/2(1.96): significance level at α =0.05 with 95% confidence interval, p: proportion of prevalence of diarrhea in eastern region which was 22.5% [25], d: margin of error (5%) and 10% non-response rate. The final required sample size was 295. Lottery method was used to select the 3 hospitals and the sample was collected proportionally from each hospital using simple random sampling method. Each hospital monthly patient flow report was used as a sampling frame.

### Operational definitions

- Dehydration: It is a condition when the child loses too much water and salt from the body [2, 26]
- Rehydration: The correction of dehydration with oral rehydration salts (ORS) or home prepared solution [2].
- Oral Rehydration Therapy (ORT): The administration of fluid by mouth to prevent or correct the dehydration that is a consequence of diarrhea. It is a mixture of clean water, salt and sugar [2].
- Good knowledge: Those mothers who answered above the mean of the knowledge questions [27].
- Poor knowledge: Those mothers who answered below the mean of the knowledge questions [27].
- Positive Attitude: Mothers who answered above the mean questions of the attitude were assigned as having “positive attitude” [28]
- Negative Attitude: those who answered below the attitude questions were assigned as having a “negative attitude” [28]
- Good practice: Mothers who able to answer above the mean of the practice questions were measured as good practice [29].
- Poor Practice: Those mothers who answer below the mean of the practice questions were measured as poor practice [29].

### Measurement and data collection procedure

Face to face interview was employed by using a standard and structured questionnaire that contained sociodemographic status, knowledge, attitude, practice, and health-seeking behavior questions of the mothers regarding under 5 children diarrheal diseases. There were four trained BSc nurse data collectors and 1 M.Sc. nurse as a supervisor.

### Data quality control

The data quality was assured by using different methods. The standard and structured questionnaire was used (Additional file 1). The questionnaire was prepared in English and translated into the local language (Amharic, oromic, and somalic) for data collection and then re translated back into English for analysis. Two days of training was given to the data collectors and supervisors on the data collection tool and procedures. Then the questionnaire was pretested on 5% of the sample size to ensure its validity. Findings from the pretesting were utilized for modifying and adjustment of the instrument and interviewing technique. Data collectors were supervised closely by the supervisors and the principal investigators. Completeness of each questionnaire was checked by the principal investigator and the supervisors on daylily basis. Double data entry was done by two data clerks and the consistency of the entered data was cross-checked by comparing the two separately entered data.

### Data processing and analysis

Immediately after the data collection was completed, each questionnaire was thoroughly reviewed for completeness and consistency by the data collectors, supervisor and investigators. Then the data were entered into Epi Info version 3.1 and analyzed using SPSS for window version 20. The descriptive statistical analysis was used to compute frequency, percentages, and mean of the findings of this study. The results were presented using tables, graphs, and result statements.

## Results

A total of 295 mothers have participated in the study with a response rate of 100%. So, 295 respondents’ data were included in the analysis process.

### Socio-demographic characteristics of the mothers

In this study, more than half of the mothers (51.5%) were in the age of 25–34 years with the mean age of 27. Based on religion, Muslims (67.5%) and Orthodox (22%) were dominant. Regarding ethnicity, 137 (46.4%) mothers were Oromo, 121 (41.0%) Somali, 31 (10.5%) Amhara and 6 (2.1%) were from other ethnicities.

From the total participants, 275 (93.2%) were married, 113 (38.3%) were housewives and 132 (44.8%) were unable to read and write. The mean monthly family income of the respondents was 1551 Ethiopian Birr. About half of the children [146 (49.5%)] were in the age group of 6–24 months (Table 1).

**Table 1:** Sociodemographic characteristics of respondents, Diredawa, East Ethiopia, 2016

Characteristic	Category	Frequency	Percentages
Age of the mother	15–24	109	36.9%
	25–34	152	51.5%
	35–44	32	10.9%
	> 45	2	0.7%
Age of the child	0–5 months	60	20.3%
	6–24 months	146	49.5%
	24–59 months	89	30.2%
Marital status of the mother	Married	275	93.2%
	Single	2	0.7%
	Widowed	6	2.0%
	Divorced/separated	12	4.1%
Occupation of the mother	Housewife	235	79.7%
	Gov’t/NGO employed	52	17.6%
	Self-employed	8	2.7%
Monthly income of the mother (Binned)	<=1000	106	35.9%
	1001–3000	148	50.2%
	3001 & above	41	13.9%
Mother’s educational status	Unable to read and write	132	44.8%
	Primary	113	38.3%
	Secondary	29	9.8%
	Diploma and above	21	7.1%
The religion of the mother	Islam	199	67.5%
	Orthodox	65	22.0%
	Protestant	29	9.8%
	Others	2	0.7%
The ethnicity of the mother	Oromo	137	46.4%
	Somali	121	41.0%
	Amhara	31	10.5%
	Others	6	2.1%

### Mothers knowledge about diarrhea prevention and management among under 5 children

Most of the mothers (92.5%), defined diarrhea as the passing of loose stool 3 or more times per day, while, only 8 (2.7%) mothers identified blood in the stool. Two hundred fifty-two (85.5%) respondents thought that diarrhea is caused by drinking contaminated water. Around half (51.2%) of the participants identified that weakness or lethargy is the danger sign of under-five diarrheal disease. To the contrary, only 2 (0.7%) of them knew that marked thirst for water is the danger sign of diarrheal disease (Table 2).

**Table 2:** Maternal knowledge about under 5 diarrheal diseases in Dire Dawa, Eastern Ethiopia, 2016

Characteristic	Frequency	%
Definition of diarrhea		
Frequent passing of watery stool (3 or more times)	273	92.5%
Frequent passing of normal stool	12	4.1%
Blood in stools	8	2.7%
Greenish stools	2	0.7%
Diarrheal causes		
Teething	15	5.1%
Evil eye	24	8.1%
Contaminated water	252	85.5%
No idea	4	1.3%
Diarrheal danger signs		
Becoming weak or lethargic	151	51.2%
Repeated vomiting/vomiting everything	103	34.9%
Fever and blood in the stool	37	12.5%
Marked thirst for water	2	0.7%
Others	2	0.7%

Regarding homemade solution, only less than half of the participants [125 (42.4%)] were used homemade solution during diarrheal disease of their child. From them, [117 (93.6%)] prepared the solution using 1/2 teaspoon of salt, and 6 teaspoons of sugar in 1 liter of water.

Around two-thirds [184 (62.4%)] of the mothers knew about the recommended volume of water for mixing a sachet of ORS (i.e., 1000 ml. of water to 1 sachet of ORS). One hundred three (34.9%) of the respondents believed that ORS should be given after the passing of every loose stool of the child, while 90 (30.4%) said that should be administered whatever child needs to drink (Table 3).

**Table 3:** Respondents’ knowledge about the correct use of ORS, Diredawa, East Ethiopia, 2016

Variable	Categories	Freq.	%
*How is ORS prepared?*	1 sachet of ORS- 300 ml (1 coke bottle) of water	25	8.5%
	1 sachet of ORS- 500 ml (1 small size of mineral bottle) of water	56	18.9%
	1 sachet of ORS- 600 ml (1 beer bottle) of water	25	8.5%
	1 sachet of ORS- 1000 ml (1 l) of water	184	62.4%
	1 sachet of ORS- 1500 ml (1.5 l or large size of mineral bottle) of water	5	1.7%
*How often should ORS be given?*	Once a day	50	17.0%
	2–3 times a day	52	17.6%
	Whatever child wants to drink	90	30.5%
	After the passing of very loose stool	103	34.9%
*How long should the mixed ORS last?*	24 h. (1 day)	255	86.4%
	48 h. (2 days)	33	11.2%
	72 h. (3 days)	4	1.4%
	96 h. (4 days)	3	1.0%

### Mother’s attitudes toward prevention and home-based management of under-five diarrhea

**Fig. 1:**
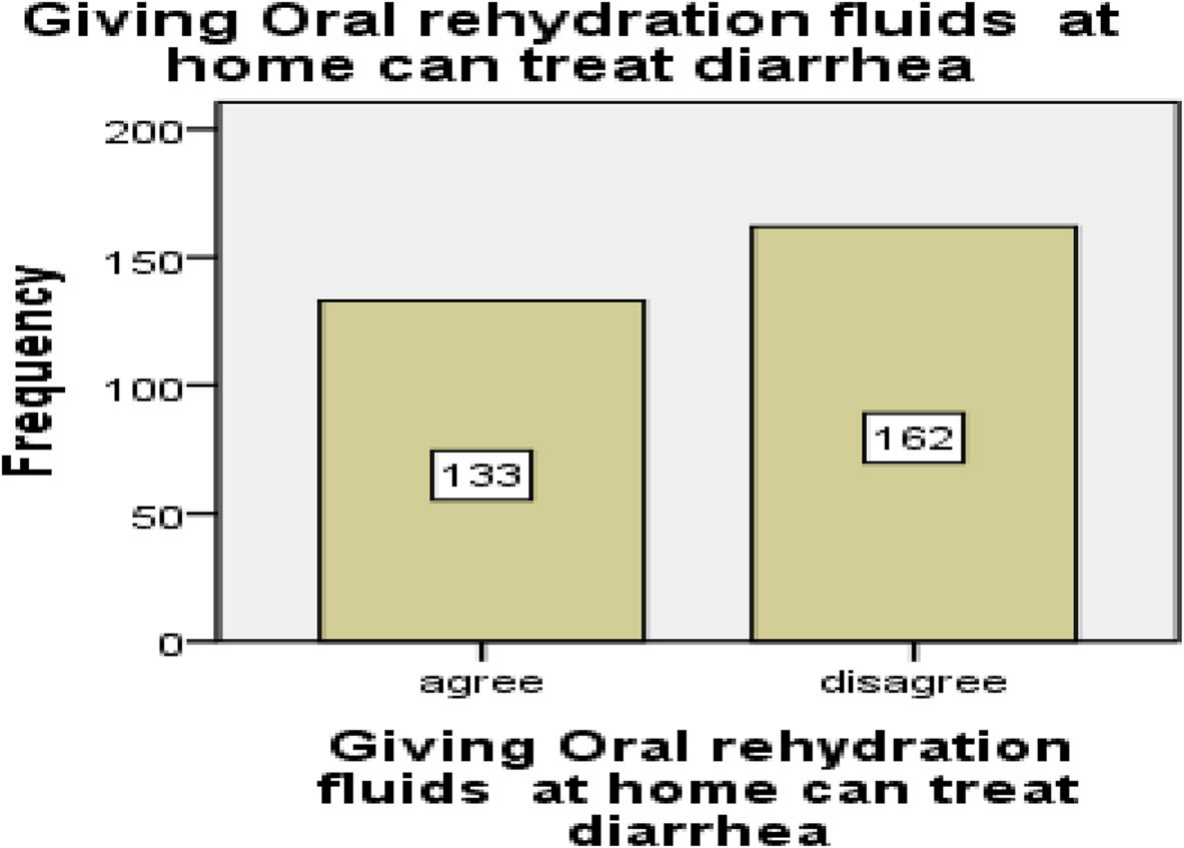
Mothers attitude toward giving oral rehydration therapy at home in Diredawa, Eastern Ethiopia, 2016

**Fig. 2:**
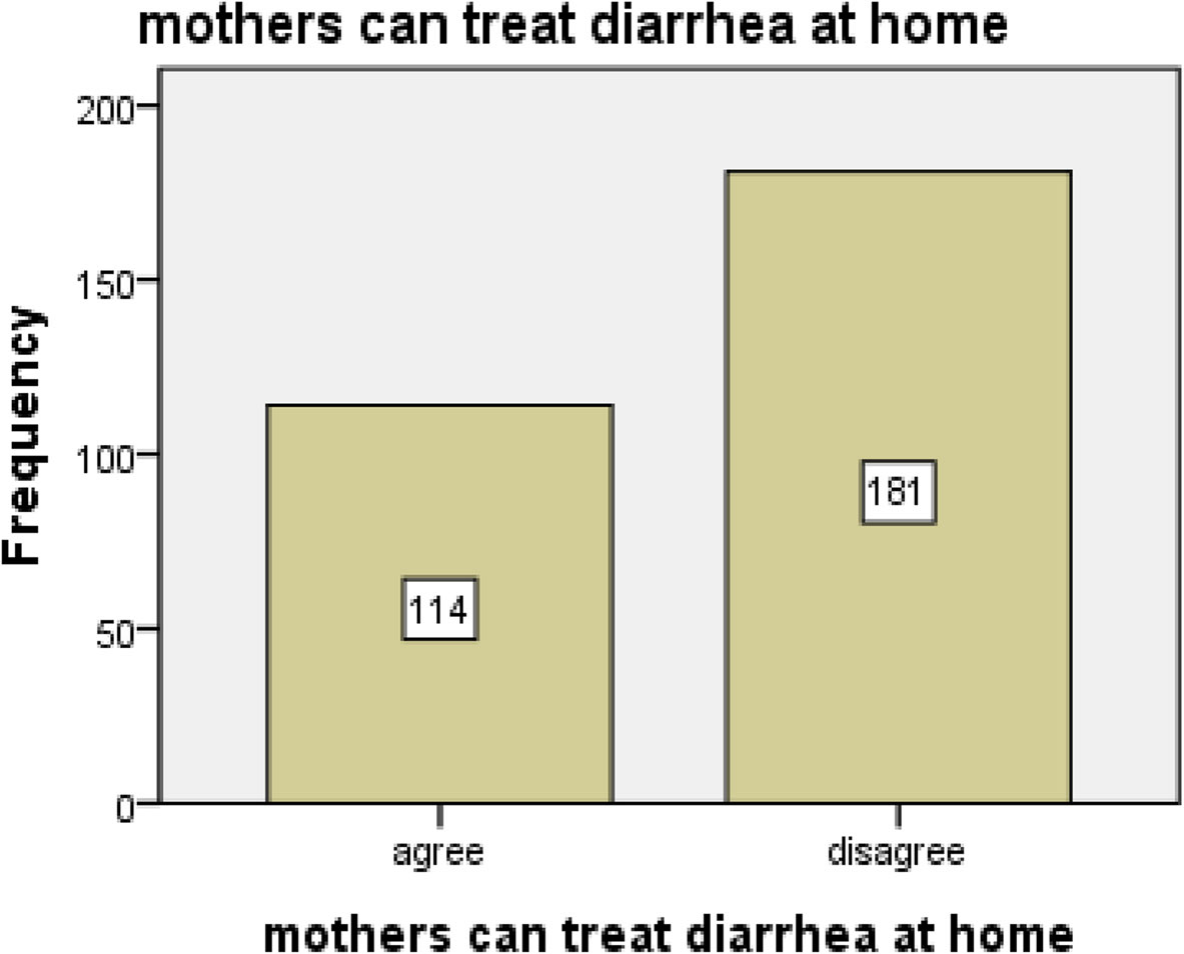
Mothers attitude towards the statement of “Mothers can treat diarrhea at home” in Dire Dawa, Eastern Ethiopia, 2016

**Fig. 3:**
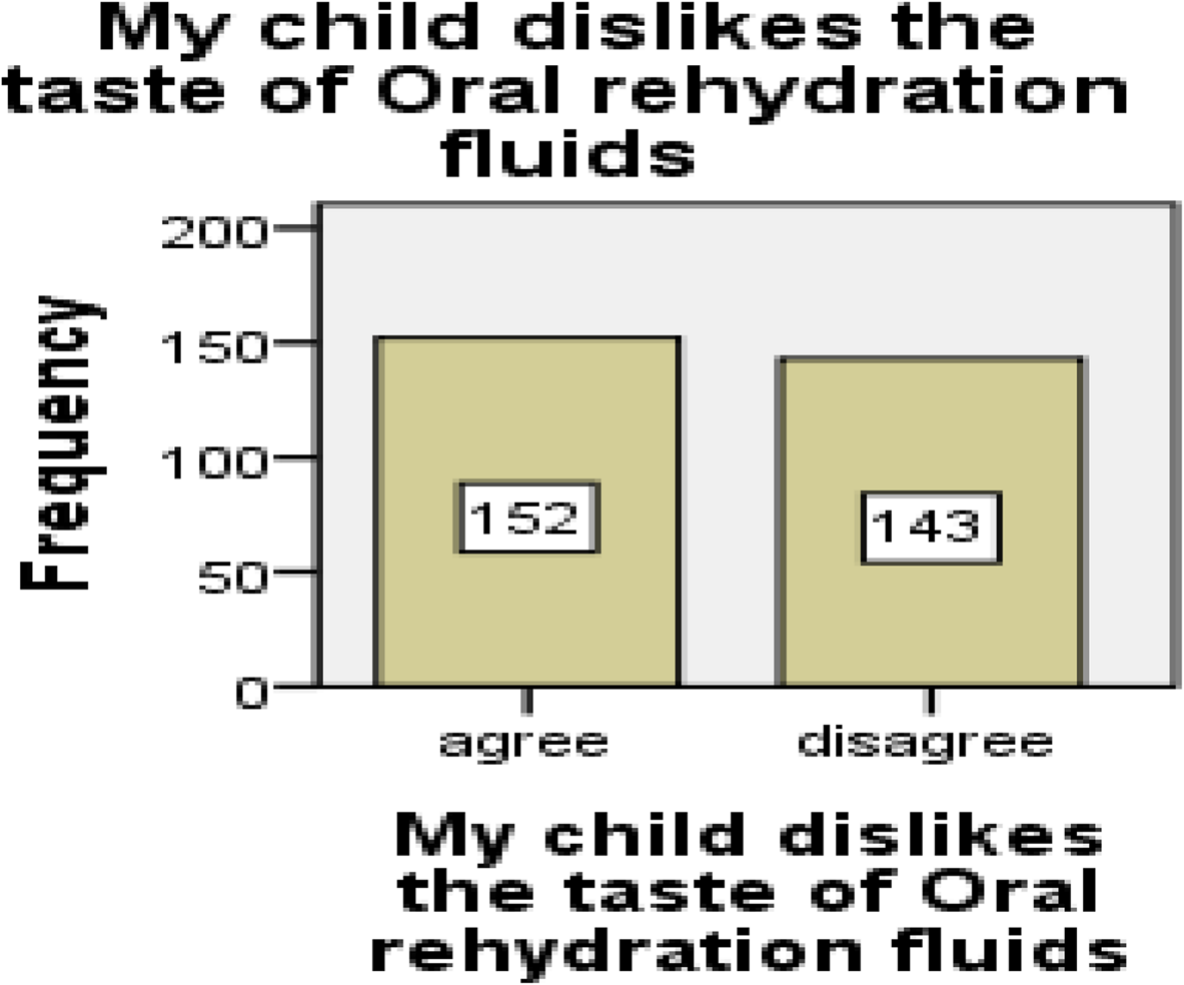
Mothers attitude about the taste of oral rehydration fluid by their children, Diredawa, Eastern Ethiopia, 2016

From the total respondents, the majority of them [162 (55%)] disagreed towards the provision of oral rehydration solution at home for the treatment of under-five diarrheal diseases. Similarly, most of the participants [181 (61.4%)] disagreed with the statement “mothers can treat their children’s diarrheal disease at home”. Around half of the mothers, 152 (51.5%) believed that their child dislikes the taste of oral rehydration solution (Figs. 1, 2, and 3).

### Practices of mothers towards the prevention and home management of diarrhea among under-five children

Only one-quarter of the mothers [77 (26.1%)] breastfed their child more than usual while majority 178 (60.3%) breastfed less than usual during the diarrheal episodes. Likewise, only 83 (28.1%) offered a drink more than usual during diarrheal episodes but most of the mothers 181 (61.4%) offered a drink for their child less than usual during the diarrheal episodes. Concerning feeding, 99 (33.6%) of mothers offered food more than usual to eat during the diarrheal episodes and 185 (62.7%) of the mother offered less than usual. Most of the mothers (67.8, 84.7% & 100%) responded that they usually wash their hands before preparing food, after preparing food, and after defecation respectively (Table 4).

**Table 4:** Maternal feeding practices during child’s diarrheal episode and hand washing behaviors in Dire Dawa, 2016

Characteristic	Category	n	%
*When (Name) had diarrhea, did you breastfeed him/her less than usual, about the same amount, or more than usual?*	Less	178	60.3%
	Same	35	11.9%
	More	77	26.1%
	Child not breastfed	4	1.4%
	Don’t know	1	0.3%
*When (Name) had diarrhea, was he/she offered less than usual to drink, about the same amount, or more than usual to drink?*	Less	181	61.4%
	Same	31	10.5%
	More	83	28.1%
	Nothing to drink	0	0.0%
	Don’t know	0	0.0%
*Was (name) offered less than usual to eat, about the same amount, or more than usual to eat?*	Less	185	62.7%
	Same	11	3.7%
	More	99	33.6%
	Nothing to eat	0	0.0%
	Don’t know	0	0.0%
*When do you wash hands with soap*	Before food preparation	200	67.8%
	Before feeding children	250	84.7%
	After defecation	295	100.0%
	Never	0	0.0%
	Other	0	0.0%

### Mothers care-seeking behavior and places during their children diarrheal episode

Almost all of the mothers [289 (98.0%)] sought medical treatment for their children during the time of diarrheal diseases. From those who sought care for their child’s diarrhea, the majority [179 (60.7%)] visited hospitals for the treatment of diarrhea, and 9 (3.1%) went to the traditional practitioner (Table 5).

**Table 5:** Mothers’ care-seeking behavior and place sought for care in Dire Dawa, Eastern Ethiopia, 2016

Characteristic	Category	n	%
*Did you seek advice or treatment from someone outside of the home for (Name’s) diarrhea?*	Yes	289	98.0%
	No	6	2.0%
*Where did you first go for advice or treatment?*	Hospital	179	60.7%
	Health center	91	30.8%
	Health post	0	0.0%
	PVO center	0	0.0%
	Clinic	16	5.4%
	Traditional practitioner	9	3.1%

### The overall level of knowledge, attitude, and practice of mothers in prevention and home-based management of diarrhea among under-five children

Knowledge was assessed by asking, whether the mothers know about ORS and what the benefits of ORS, and so on. Mothers who respond above the mean of the questions correctly were assigned as having “good knowledge” while mothers who answered below the mean were regarded as having “poor knowledge”:

Also, the attitude was assessed whether they agree or disagree towards the taste of ORS to their child, or whether they agree or disagree that ORS is the first choice in the management of diarrhea and so on. Mothers who answered above the mean questions were assigned as having “positive attitude” and those who answer below the mean were assigned as having “negative attitude”.

Like others, the overall practice of mothers was measured by asking how is ORS prepared, how often is it given and how long should a mixed ORS last and so on. Mothers who answered above the mean questions were assigned as having “good practices” whereas those who did not be assigned as having “poor practice”.

Based on these criteria, 192 (65.2%) of the mothers had good knowledge and 103 (34.9%) had poor knowledge about the prevention and home-based management of under 5 diarrheal diseases. Regarding the attitude, more than half of the mothers (54.9%) had a negative attitude and only 133 (45.1%) had a positive attitude towards the prevention and home-based management of under 5 diarrheas. From the total of mothers participated in this study, only 124 (42%) of them had a good practice and the remaining 171 (58%) had poor practice towards prevention and home-based management of under 5 diarrheas.

## Discussion

This study has assessed mothers’ knowledge, attitude, and practices towards the prevention and home-based management of under 5 diarrheal diseases in Diredawa city, Eastern Ethiopia. Based on the findings, the majority of the respondents (65.2, 54.9, and 58%) had good knowledge, negative attitude and poor practice about the prevention and home-based management of under 5 diarrheal diseases respectively.

The finding of this study showed that 65.2% of mothers had a good knowledge about prevention and home-based management of diarrhea among under-five children. A similar finding was observed in Fenoteselam, Ethiopia (65.9%) [29]. On the contrary, this finding is higher than studies done in Kashan, Iran (28.8%), Fagita Lekoma, Ethiopia (56.2%), and Assosa, Ethiopia (37.5%) [27, 28, 30]. This is mainly due to the fact that Dire Dawa city is a bigger and more urbanized city with many mass media.

Most of the mothers (92.2%) defined diarrhea correctly (as the passing of loose stool 3 or more times per day); which is much higher than other studies done in Fagita Lekoma, Ethiopia (65.4%), Karachi, Pakistan (52.5%) [24, 27]. Similarly, in this study, two hundred fifty-two (85.5%) respondents thought that diarrhea is caused by drinking contaminated water; that is significantly higher than studies conducted in Pakistan, India, Mali, and Western Ethiopia [24, 28, 31, 32]. The probable explanation of the discrepancy might be due to the presence of many mass media and health facilities in the city, which may disseminate information to the population and create good knowledge towards under-five diarrheal diseases.

Less than half of the participants (42.4%) were used homemade solution during diarrheal disease of their child. The result different from the Heidedal community (90%), Taung district (83.6%), Swaziland community (97%) of South Africa [33]. This might be due to the fact that most of the mothers in the city sought medical treatment for their children during the time of diarrheal diseases.

Around two-thirds [184 (62.4%)] of the mothers knew about the recommended volume of water for mixing a sachet of ORS. This is much less than other studies done in Ethiopia (85.4%), Pakistan (75.5%), Nepal (70%), and India (76.7%) [24, 27, 31, 34]. This could be justified by the fact that these mothers might not be familiar with ORS mixing due to lack of education.

Also, the majority of the mothers agreed that ORT can replace lost fluid but they disagreed ORT is the first-choice management of diarrhea. Similarly, a study done in Mali showed that majority of mothers knew ORT can replace lost fluid but its inability to stop diarrhea caused them to seek additional treatments such as antibiotics and traditional medicines to treat diarrhea [32].

This study indicated that 42% of mothers had good practice in prevention and home-based management of diarrhea. This is compiled with the finding of Northwest, Ethiopia (44.9%), but the opposite was observed in studies conducted in Assossa District (62.9%) and Awi zone (37.6%), [27–29]. The difference may be due to the difference of the study area, period and sample size.

In this study, 61.4 and 62.7% of the mother offered fluid and feeding less than usual to their child during the diarrheal episodes respectively. In the same way, more than 70% of mothers in Kenya and 19.6% of mothers in India decrease fluid intake and feeding during the diarrheal episodes [31, 35]. To the contrary, other studies in Bangladesh and Pakistan showed that more than 50 and 71% of mothers were in favor of giving food and fluids during the diarrheal illness of the child [24, 36]. Majority of the mothers in this study area were uneducated and this might be the major reason for the discrepancy as uneducated mothers could not have the opportunity to get information from books, newspaper, and other reading sources. The other possible reason for the decrement of fluid intake and feeding during diarrheal illness by the mothers might be due to the fear of more vomiting and lose of watery stool.

Most of the mothers (67.8% & 100%) usually wash their hands before preparing food, and after defecation respectively. But in Assossa, Ethiopia only 11.7, and 16%, of the mothers was wash their hands before preparing food, and after defecation respectively [28]. To contrary, in Bangladesh, 60.0 and 3.1% don’t wash their hands before food preparation and after defecation respectively [36]. This variation might be due to differences in culture, sociodemographic and information access.

Almost all of the mothers [289 (98.0%)] in the present study sought medical treatment for their children during the time of diarrhea diseases which much different from Fagita Lekoma, Ethiopia (71.6%), Karachi, Pakistan (52.5%) and Assossa, Ethiopia (62.4%) [24, 27, 28]. As Diredawa is a highly urbanized city, mothers have more opportunity to access health facilities within the near distance.

## Conclusions

The finding of this study showed that the attitude and practice of mothers were unsatisfactory about the prevention and home-based management of under-five diarrheal diseases. Therefore, Health education, dissemination of information, and community conversation should plan and implement to create a positive attitude and practice towards the better prevention and management of under 5 diarrheal diseases.

## Strength and limitation of the study

As there was no the same study in the study area, it can use as a baseline for other studies. Similarly, it can also be a blueprint to conduct an interventional study in the particular area.

The limitation of this study is that it was not possible to establish a temporal relationship between the exposure and outcome variable as this study design was a cross-sectional study. Additionally, determinant factors for the negative attitude and poor practice of the mothers were not included due to the limitation of time and resource. So, another study is needed to determine these associated factors.

## Additional file

Additional file 1: English language copy of the questionnaire. (DOCX 30 kb)

## Abbreviations

EDHS: Ethiopian Demographic and Health Survey
EPI: Expanded Program on Immunization
FMOH: Federal Ministry of Health
HIV: Human Immunodeficiency Virus
IMNCI: Integrated Management of Neonatal and Childhood Illnesses
IV: Intra-venous
Kg: Kilograms
MDG: Millennium Development Goal
Ml: Milliliters
ORS: Oral rehydration salt
ORT: Oral Rehydration Therapy
RHFs: Recommended Home Fluids
SPSS: Statistical Package for Social Science
SSS: Sugar Salt Solution
SSW: Sugar-Salt- Water
UNICEF: United Nations International Children Emergency Fund
WHO: World Health Organization
