# Supplementary material for: Mothers’ knowledge, attitude and practice towards the prevention and home-based management of diarrheal disease among under-five children in Diredawa, Eastern Ethiopia, 2016: a cross-sectional study
Source: BMC Pediatr. 2018 Nov 19;18:358. doi: 10.1186/s12887-018-1321-6 (PMC6241041; doi:10.1186/s12887-018-1321-6)
Supplement: Supplementary file 1 — English language copy of the questionnaire. (DOCX 30 kb) [file 12887_2018_1321_MOESM1_ESM.docx]

**Additional file 1**

**QUESTIONNAIRE (ENGLISH)**

**HARAMAYA UNIVERSITY**

**COLLEGE OF HEALTH AND MEDICAL SCIENCE**

**DEPARTMENT OF NURSING**

This questionnaire is designed to collect information from respondents in respects of this study.

Hello, my name is____________________ I am here today to collect data by this interview regarding about KAP of mothers in prevention and home-based management of diarrhea in under five children.

You are among those chosen to participate in the study. Thus, the investigator of this research requests you for information and it appreciates your willingness to support its efforts. We believe that you will find this questionnaire interesting and we looking forward to receiving your response. All information obtained from you will be used only for the purpose of this research. Both yours and your Child’s name will not be written in this form and will never be used in connection with any information you tell us.

**Are you willing to participate?**

1. **Yes B. No**

**PART I. SOCIOECONOMIC STATUS**

| S.N | QUESTIONS | RESPONSES |
| --- | --- | --- |
|  | Age of mother |  |
|  | Age of child |  |
|  | Marital status | A. Married B. single  C. Widowed D. divorced/separated |
|  | Occupation of mother | A. Housewife B. Employed ( both governmental or nongovernmental) C. Self employed |
|  | How much is your family monthly income? | ________Ethiopian Birr |
|  | Educational status mother | A. Unable to write and read  B. primary C. secondary  D. diploma and above |
|  | Religion | A. Muslim B. Orthodox  C. Protestant D. Other (specify) |
|  | Ethnicity | A.Oromo.B)Somali(C).Amhara D.Other (specify).............. |

**PART II. KNOWLEDGE OF MOTHER ABOUT DIARRHEA PREVENTION AND HOME BASED MANAGEMENT**

| ***S.NO*** | ***QUESTIONS*** | | ***RESPONSES*** | ***SKIP*** |
| --- | --- | --- | --- | --- |
| 1 | What do you understand by diarrhea? | | 1. Frequent passing of watery stool (3 or more times) 2. Frequent passing of non-watery stool 3. Blood in stools 4. Greenish stools 5. No idea 6. Others, please specify………….. |  |
| 2 | What do you think are the causes of diarrhea? | | 1. Teething 2. Evil eye 3. Contaminated water 4. No idea 5. Others, please specify ……….. |  |
| 3 | What are some of the danger signs associated with diarrhea? | | 1. Becoming weak or lethargic Frequent passing of diarrhea 2. Repeated vomiting/vomiting everything 3. Fever and blood in the stool 4. Marked thirst for water 5. Others, please specify …………… |  |
| 4 | Do you know how to prevent diarrhea from your child | | A: YES B: NO |  |
| 5 | Do you know what oral rehydration therapy (ORT) is used for? | | 1. YES B.NO |  |
| 6 | If yes, What are the use of oral rehydration therapy (ORT) | | A. Adds the lost water to the child’s body  B. cure diarrheal disease  C. no idea  D. Others_____(specify) |  |
| 7 | Have you ever used oral rehydration therapy (ORT) | 1. YES B. NO | |  |
| 8 | Do you know how to prepare oral rehydration therapy (ORT) at home? | 1. YES B. NO | |  |
| 9 | If yes how do you prepare it? | A. 1 teaspoon of salt, 8 teaspoons of sugar in one liter of water.  B. other give formula | |  |

| **10** | How do you give ORS to your child | A. after every watery stool  B. once a day  C.2-3 times a day  D. wherever the child wants to drink |  |  |  |
| --- | --- | --- | --- | --- | --- |
| **11** | How long should the mixed ORS last? | 1. 24 hrs (I day) 2. 48 hrs (2days) 3. 72 hrs (3days) 4. 96 hrs (4days)   Others please specify ………………… |  |  |  |

**PART III. ASSESSMENT OF ATTITUDE OF MOTHERS TOWARD PREVENTION AND HOME BASED MANAGEMENT OF DIARRHEA IN THEIR UNDER FIVE CHILDREN**

| 1 | Diarrhea is preventable disease and It is manageable at home | 1. AGREE   2. DISAGREE |  |
| --- | --- | --- | --- |
| 2 | Oral rehydration fluids are the first-line treatment of diarrhea in children | 1.AGREE  2.DISAGREE |  |
| 3 | Mothers can prepare oral rehydration at home | 1.AGREE  2.DISAGREE |  |
| 4 | My child dislikes the taste of Oral rehydration fluids | 1.AGREE  2.DISAGREE |  |
| 5 | Giving Oral rehydration fluids at home can treat diarrhea | 1.AGREE  2.DISAGREE |  |
|  |  |  |  |
| 6 | Oral rehydration fluids replace the fluids lost in diarrhea | 1.AGREE  2.DISAGREE |  |

**PART IV. ASSESSMENT OF PRACTICE OF MOTHERS IN PREVENTION AND HOME BASED MANAGEMENT OF DIARRHEA IN THEIR CHILDREN**

| 1 | | Do you prepare oral rehydration therapy (ORT) at home | | 1. YES 2. NO |  |
| --- | --- | --- | --- | --- | --- |
| 2 | | How is ORS prepared? | | 1. 1 sachet of ORS- 300 mls (1 coke bottle) of water1 2. 1 sachet of ORS- 500 mls (1 small size of mineral bottle) of water 3. 1 sachet of ORS- 600 mls (1 beer bottle) of water 4. 1 sachet of ORS- 1000 mls (1 liter) of water 5. 1 sachet of ORS- 1500mls (1.5 liters or large size of mineral bottle) of water 6. Other(specify)…….. |  |
| 3 | | Where did you seek help for your Diarrheal child? | | 1. Herbalist 2. Magician 3. Private drug-shop 4. Health center 5. Hospital 6. Another place |  |
| 4 | | When (NAME) had diarrhea, did you breastfeed him/her less than usual, about the same amount, or more than usual? | | LESS 1  SAME 2  MORE 3  CHILD NOT BREASTFED 4  DON’T KNOW 5 |  |
| 5 | | When (NAME) had diarrhea, was he/she offered less than usual to drink, about the same amount, or more than usual to drink? | | LESS 1  SAME 2  MORE 3  NOTHING TO DRINK 4  DON’T KNOW 5 |  |
| 6 | | Was (NAME) offered less than usual to eat, about the same amount, or more than usual to eat? | | LESS 1  SAME 2  MORE 3  NOTHING TO EAT 4  DON’T KNOW 8 |  |
| 7 | | Did you seek advice or treatment from someone outside of the home for (NAME’S) diarrhea? | | YES 1  NO 2 |  |
| 8 | | Where did you first go for advice or treatment? | | **HEALTH FACILITY**  HOSPITAL 01  HEALTH CENTER 02  HEALTH POST 03  PVO CENTER 04  CLINIC 05  FIELD/COMMUNITY HEALTH  WORKER 06  OTHER HEALTH  FACILITY**____________________** 07  (SPECIFY)  **OTHER SOURCE**  TRADITIONAL PRACTITIONER 08  SHOP 09  PHARMACY 10  COMMUNITY DISTRIBUTORS 11  FRIEND/RELATIVE 12  OTHER_________________________ 13  (SPECIFY) |  |
| 9 | | Does your household have a special place for hand washing? | | YES 1  NO 2 |  |
| 10 | When do you usually wash your hands with soap/ash?  RECORD ALL MENTIONED. | | A: NEVER B:BEFORE FOOD PREPARATION C:BEFORE FEEDING CHILDREN D:AFTER DEFECATIONE:AFTER ATTENDING TO A CHILD WHO HAS DEFECATED  F:OTHER__________________________  (SPECIFY) | |  |

***Thank you***
